# Investigating Agile Adoption in Saudi Arabian Mobile Application Development

**DOI:** 10.1007/978-3-030-58858-8_27

**Published:** 2020-08-18

**Authors:** Fahad S. Altuwaijri, Maria Angela Ferrario

**Affiliations:** 6 IT University of Copenhagen, Copenhagen, Denmark; 7 University of British Columbia, Vancouver, BC Canada; 8 School of Computing and Communications, Lancaster University, Lancaster, UK; 9 Department of Information Technology, College of Computer, Qassim University, Buraydah, Saudi Arabia

**Keywords:** Agile software development, Agile methods, Agile, Mobile application development, Mobile apps, Software engineering

## Abstract

Mobile app development has been considered as one of the fastest growing segments of the software industry both worldwide and in Saudi Arabia. Due to their pervasiveness, mobile applications call for consideration of complex and rapidly changing requirements given the diversity of their environments. Therefore, agile is considered the most suitable methodology for developing mobile apps. However, little research has investigated agile adoption in mobile app development in the real context. Therefore, the purpose of this PhD is to investigate the factors that have a significant impact on agile adoption in mobile app development by small and medium-size software organisations in Saudi Arabia. The expected key contribution of this research will be a deep insight into agile adoption in mobile app development, and the design and development of tools and techniques that may support agile adoption within Saudi context.

## Introduction

The aim of this PhD research is to investigate the factors influencing agile adoption in mobile application development sector in Saudi Arabia. Mobile app development has been considered as one of the fastest growing segments of the software industry both worldwide [1], and in Saudi Arabia [2] with mobile devices now becoming integral parts of our lives across domains such as health, entertainment, education and marketing. Due to their pervasiveness and ubiquity, mobile applications call for careful consideration of complex and rapidly changing requirements given the diversity of the environments of their use in terms of user experience, user interface, and reception quality [3, 4].

Although there have been several studies that concluded that agile is a natural fit for mobile app development [4–7], there is a need for empirical evidence-based research that investigates the specific factors (e.g. cultural, technical and environmental) that support or challenge the agile adoption in mobile app development by small and medium-size software organisations. To the best of the author’s knowledge, there are no studies about that in Middle Eastern countries, particularly in Saudi Arabia.

In the following subsections, the research aims and objectives are presented as well as the research questions. Section 2 briefly summarises the related work. The research methodology design is provided in Sect. 3, covering a description of each step of the research process. Section 4 discusses the validity threats. The last section outlines the current status of my PhD and some future works.

### Research Aims and Objectives

This research aims to investigate the key factors that can either support or hinder agile adoption in mobile app development by software organisations in the Kingdom of Saudi Arabia. It is intended that the key research contribution will be twofold: (1) a deeper insight into agile adoption in Saudi mobile app development; and (2) the development of tools and techniques that support agile work in Saudi Arabia. These aims will be achieved through the following objectives:

1. Review current literature about the adoption of agile, particularly in mobile app development.
2. Investigate the awareness, current usage, and perception of agile by Saudi mobile app practitioners through empirical research.
3. Obtain a deeper insight into the factors that may influence agile adoption in Saudi Arabian mobile app development through empirical research.
4. Design and develop tools and techniques that can support agile adoption in Saudi Arabia’s mobile app industry.

### Research Questions

The main research questions that motivated this research are:

**RQ1. What are the types of factors that support or hinder agile adoption in mobile app development in the context of Saudi Arabian software organisations?**

The main research question is divided into four sub-research questions:

- ***RQ1.1*** *What is the state-of-art research in agile adoption in general and particularly its adoption in mobile app development?*
- ***RQ1.2*** *What is the level of awareness of agile amongst mobile app practitioners in Saudi Arabia? How is it perceived?*
- ***RQ1.3*** *What are the enabling factors and the challenges of adopting agile in mobile app development in the context of Saudi Arabia?*
- ***RQ1.4*** *What are the mechanisms (including software tools and techniques) that can support agile adoption in Saudi Arabian mobile app development?*

## Related Work

### Agile Software Development Adoption

It is important to investigate the facilitating factors and the challenges related to the adoption of agile principles and practices in developing software projects. This is because such understanding will help determine to what extent agile can be adopted and how it influences the success of projects. In this regard, scholars have advocated that the suitability of agile adoption by software organisations depends on the practitioners’cultural background, hence, agile is dependent on several human factors [8, 9]. Studies have found that practitioners’ culture, communication, skills and experiences are considered as the most important factors that influence the adoption of agile [10, 11]. Furthermore, organisational aspects are considered as one of the most significant aspects of agile adoption [9, 11]. On the other hand, Chow and Cao [8] argued that besides the importance of organisational and people aspects, technical factors have a significant impact on agile adoption, including the agile software techniques and delivery strategies.

All of the studies mentioned above advocate that the practice of agile is mainly influenced by human factors. This means that people or organisations in different countries practice agile differently according to their cultural differences. Therefore, this research will investigate the factors identified in previous studies to determine whether they can be considered as the main aspects affecting the adoption of agile in Saudi mobile app development. Although there are numerous studies that focused on identifying the factors influencing agile adoption [10, 12, 13], there is a lack of studies on the adoption of agile in Middle Eastern countries, particularly its adoption in mobile app development in Saudi Arabia. With regards to investigating agile adoption in mobile app development, several studies have focused on identifying the benefits and challenges of the adoption and discussing the proposed agile-based mobile methodologies such as Mobile-D [5, 6]. However, these studies did not investigate the factors influencing agile adoption in mobile app development.

### Agile Awareness and Perceptions

The initial step in investigating the factors influencing agile adoption by software organizations is to examine practitioners’ awareness and perceptions of agile. Several research efforts about this topic, however, most of these studies were conducted in developed countries such as [14–16] and only a handful were conducted in the context of developing countries such as Brazil [17], Paraguay [18] and India [19]. Unfortunately, none of these studies is focused on agile perceptions and usage in mobile app development in the Middle Eastern countries, especially Saudi Arabia. In the context of Saudi Arabia, Bin-Hezam et al. [20] studied to what extent agile has been adopted by SMEs in Saudi Arabia. This study was applied to different enterprises (i.e. technical and non-technical) and did not target mobile software organisations.

Some existing research examined the awareness and perception on a global scale. An example is the work of Begel and Nagappen [14] who investigated that among Microsoft employees. On the other hand, even though this study was considered global because the data was collected from three continents (i.e. North America, Asia and Europe), it only concentrated on one company that has similar aspects across the world. Therefore, to the best of the author’s knowledge, there has been no study about the level of awareness among Saudi mobile app developers towards agile, the reasons for agile adoption and non-adoption from their point of view, their perceptions towards agile methods and the tools and techniques used to support their agile teams and their limitations.

## Research Methodology

The design of this research will be explorative and inspired by interdisciplinary research framework [21], which is agile, people-focused and reflective. Using an agile approach in managing our PhD research will help us move forward quickly and reflectively through the research process. Hence, the results from each study will be used to inform and shape the subsequent studies of the research.

### Empirical Investigation Design

**Fig. 1.**
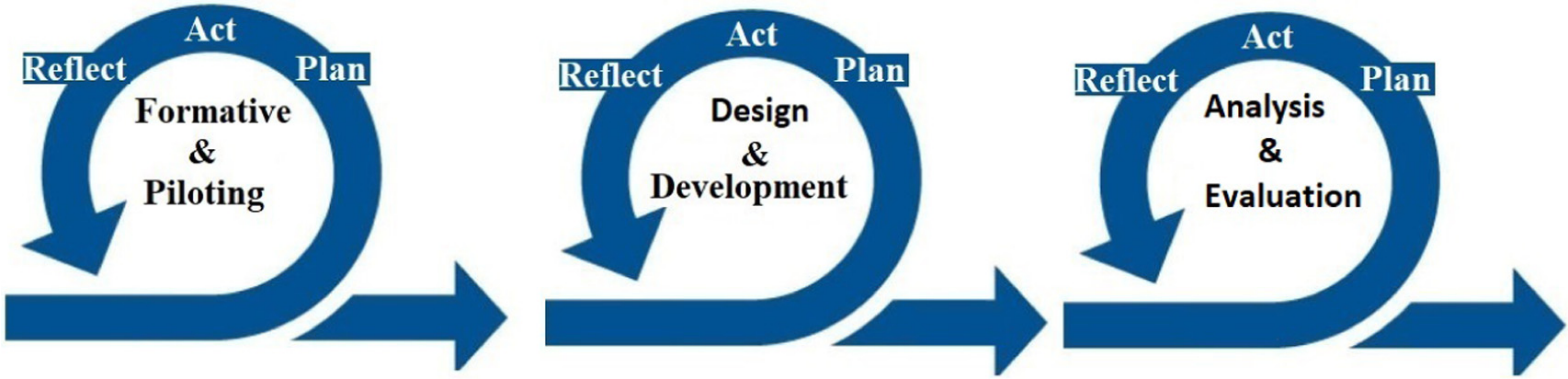
Research cycles

This research is divided into three cycles, which are explained below and summarised in Fig. 1. Each cycle will last for 7–9 months and involves three iterative stages (i.e. plan, act and reflect). In each cycle, there are several sprints each of which will last for 2–4 weeks.

**The First Cycle: Formative and Piloting.** This cycle aims to study the current related work and to understand the current usage and perception of agile in Saudi Arabia. Expert interviews will be conducted to take the experts’ viewpoint about the perception of agile and take their opinions before designing next studies. In addition, a survey questionnaire will be conducted to identify the awareness and perception of software development in general, particularly agile among Saudi mobile app developers who either adopt or do not adopt agile methods.

**The Second Cycle: Design and Development.** This cycle aims to conduct in-depth investigation to obtain a deep insight into the key factors that may influence agile adoption in Saudi mobile app development and the tools and techniques used. This investigation will be achieved through three data collection methods (i.e. interviews, observation and a focus group). The results of each activity will be used to inform and shape the next one. In addition, a prototype of tools or techniques that can support agile team within Saudi context will be designed and developed. If there are certain tools and techniques that widely acceptable in agile in western context, but may not be suitable in Saudi context, we will investigate what mechanisms could support the outcome of these tools and techniques in Saudi context.

**The Third Cycle: Analysis and Evaluation.** This cycle aims to analyse and evaluate the factors and tools, as well as to conclude the writing up of the thesis. A questionnaire will be utilised in this study to analyse the relationships between variables with a statistical technique (i.e. Factor Analysis). In terms of the tools and techniques developed, they will be evaluated based on the interviews with the agile team members who will use them.

### Data Analysis

The data collected from the quantitative methods will be analysed using a statistical software (i.e. SPSS). This will determine the relationships and trends in the data and illustrate them through graphs and cross-tabulated formats. In addition, Factor Analysis (FA) will be used to analyse the relationships between variables [22]. With regards to the data collected from qualitative methods, NVivo software will be used for organising and coding the data. In addition, the data will be subjected to the approach of thematic analysis that helps in developing themes and patterns from the data collected [23].

## Validity Threats and Control

The validity threats are discussed in this research to explain how to reduce these threats. Using the empirical research method, I will reduce my bias by applying mixed research methods as different data collection methods will be used. A pilot test for each data collection method will be conducted to avoid the threat of having questions that can be hard to understand by the participants. In terms of the research context, the study will not be limited to a specific software organisation and data will be collected from different teams from different organisations to represent organisations throughout Saudi context. Furthermore, my supervisor has strong experience in empirical research methods, thus, she could be a reference point to ensure the validity of the study.

## Current Status

This research is still in the early phase, thus, we have not started the fieldwork yet. Several tasks have been completed over the last months. First, we have reviewed the current literature. Second, we have designed the research methods that will be used throughout this research. Third, we have contacted mobile app developers in Saudi Arabia to participate in our study, and they agreed to collaborate with us. Finally, we have designed the first empirical study (i.e. expert interviews) that is seeking approval from the ethics committee. The next step will be conducting expert interviews. A survey questionnaire will be designed and shaped based on the finding of the expert interviews to the awareness and perceptions of agile. After that, we will begin to investigate the key factors influencing agile adoption through empirical research.

## References

[1] Ahmad A, Li K, Feng C, Asim SM, Yousif A, Ge S (2018). An empirical study of investigating mobile applications development challenges. IEEE Access.

[2] Ernst & Young Global Limited. Unlocking the digital economy potential of the Kingdom of Saudi Arabia, Technical report, Ernst & Young Global Limited (2019)

[3] Aldayel, A., Alnafjan, K.: Challenges and best practices for mobile application development: review paper. In: ACM International Conference on Compute and Data Analysis Proceeding Series, vol. Part F1302, pp. 41–48 (2017)

[4] Wasserman, T.: Software engineering issues for mobile application development. In: Proceedings of the ACM Workshop on the Future of Software Engineering Research FoSER, pp. 397–400 (2010)

[5] Corral, L., Sillitti, A., Succi, G.: Software development processes for mobile systems: is agile really taking over the business? In: 2013 IEEE 1st International Workshop on the Engineering of Mobile-Enabled Systems (MOBS), pp. 19–24. IEEE (2013)

[6] Kaleel, S.B., Harishankar, S.: Applying agile methodology in mobile software engineering: android application development and its challenges. Computer Science Technical Reports, pp. 1–11 (2013)

[7] Francese, R., Gravino, C., Risi, M., Scanniello, G., Tortora, G.: Mobile app development and management: results from a qualitative investigation. In: Proceedings - 2017 IEEE/ACM 4th International Conference on Mobile Software Engineering and Systems MOBILESoft 2017, pp. 133–143 (2017)

[8] Chow T, Cao DB (2008). A survey study of critical success factors in agile software projects. J. Syst. Softw..

[9] Misra SC, Kumar V, Kumar U (2009). Identifying some important success factors in adopting agile software development practices. J. Syst. Softw..

[10] Cockburn A, Highsmith J (2001). Agile software development: the people factor. IEEE Comput..

[11] Gandomani TJ, Nafchi MZ (2016). Agile transition and adoption human-related challenges and issues: a grounded theory approach. Comput. Hum. Behav..

[12] Conboy K, Coyle S, Wang X, Pikkarainen M (2011). People over process: key challenges in agile development. IEEE Softw..

[13] Iivari J, Huisman M (2007). The relationship between organizational culture and the deployment of systems development methodologies. Mis Q..

[14] Begel, A., Nagappan, N.: Usage and perceptions of agile software development in an industrial context: an exploratory study. In: The First IEEE International Symposium on Empirical Software Engineering and Measurement (ESEM), pp. 255–264 (2007)

[15] Pikkarainen M, Salo O, Kuusela R, Abrahamsson P (2012). Strengths and barriers behind the successful agile deployment-insights from the three software intensive companies in Finland. Empirical Softw. Eng..

[16] Rodríguez, P., Markkula, J., Oivo, M., Turula, K.: Survey on agile and lean usage in finnish software industry. In: International IEEE Symposium on Empirical Software Engineering and Measurement, pp. 139–148 (2012)

[17] de O. Melo, C., et al.: The evolution of agile software development in Brazil. J. Braz. Comput. Soc. **19**(4), 523–552 (2013). 10.1007/s13173-013-0114-x

[18] Salinas MRN, Neto AGSS, Emer MCFP, Santos VA, Pinto GHL, Serra Seca Neto AG (2018). Concerns and limitations in agile software development: a Survey with Paraguayan Companies. Agile Methods.

[19] Nazir, N., Hasteer, N., Bansal, A.: A survey on agile practices in the Indian IT industry. In: Proceedings of the 2016 6th International Conference - Cloud System and Big Data Engineering, Confluence 2016, pp. 635–640 (2016)

[20] Bin-Hezam, R., Bin-Essa, A., Abubacker, N.F.: Is the agile development method the way to go for small to medium enterprises (SMEs) in Saudi Arabia? In: 21st IEEE Saudi Computer Society National Computer Conference NCC 2018, pp. 1–6. IEEE (2018)

[21] Ferrario, M.A., Simm, W., Newman, P., Forshaw, S., Whittle, J.: Software engineering for ’social good’: Integrating action research, participatory design, and agile development. In: Companion Proceedings of the 36th International Conference on Software Engineering, pp. 520–523. Association for Computing Machinery (2014)

[22] Field A (2013). Discovering Statistics Using IBM SPSS statistics.

[23] Boyatzis, R.: Thematic Analysis and Code Development. Sage Publications Inc. (1998)

